# DFT Calculation of Carbon-Doped TiO_2_ Nanocomposites

**DOI:** 10.3390/ma16186117

**Published:** 2023-09-07

**Authors:** Kim Robert Gustavsen, Tao Feng, Hao Huang, Gang Li, Urszula Narkiewicz, Kaiying Wang

**Affiliations:** 1 Department of Microsystems, University of South-Eastern Norway, 3184 Horten, Norway; kim.r.gustavsen@usn.no (K.R.G.); hao.huang@usn.no (H.H.); 2 Institute of Energy Innovation, College of Materials Science and Engineering, Taiyuan University of Technology, Taiyuan 030024, China; fengtao22657@163.com (T.F.); ligang02@tyut.edu.cn (G.L.); 3 Department of Inorganic Chemical Technology and Environment Engineering, Faculty of Chemical Technology and Engineering, West Pomeranian University of Technology in Szczecin, 70-322 Szczecin, Poland; urszula.narkiewicz@zut.edu.pl

**Keywords:** TiO_2_, carbon doping, DFT, DOS, free energy

## Abstract

Titanium dioxide (TiO_2_) has been proven to be an excellent material for mitigating the continuous impact of elevated carbon dioxide concentrations. Carbon doping has emerged as a promising strategy to enhance the CO_2_ reduction performance of TiO_2_. In this study, we investigated the effects of carbon doping on TiO_2_ using density functional theory (DFT) calculations. Two carbon doping concentrations were considered (4% and 6%), denoted as TiO_2_-2C and TiO_2_-3C, respectively. The results showed that after carbon doping, the band gaps of TiO_2_-2C and TiO_2_-3C were reduced to 1.58 eV and 1.47 eV, respectively, which is lower than the band gap of pure TiO_2_ (2.13 eV). This indicates an effective improvement in the electronic structure of TiO_2_. Barrier energy calculations revealed that compared to pure TiO_2_ (0.65 eV), TiO_2_-2C (0.54 eV) and TiO_2_-3C (0.59 eV) exhibited lower energy barriers, facilitating the transition to *COOH intermediates. These findings provide valuable insights into the electronic structure changes induced by carbon doping in TiO_2_, which can contribute to the development of sustainable energy and environmental conservation measures to address global climate challenges.

## 1. Introduction

The increasing concentration of CO_2_ in the Earth’s atmosphere is one of the most pressing challenges facing humanity today, with profound implications for climate change and global sustainability. Earth’ temperature could be 33 °C lower without the greenhouse effect, and carbon dioxide makes up about 76% of the greenhouse gases [1,2,3,4,5]. To mitigate the impact of CO_2_ emissions, there is a growing interest in exploring novel materials for efficient CO_2_ capture, storage, and utilization (CCU). Among these materials, titanium dioxide (TiO_2_) has shown great promise due to its abundance, low cost, and environmental friendliness. However, the practical application of TiO_2_ is limited by its moderate CO_2_ adsorption capacity and relatively large energy band gap. In recent years, efforts have been made to improve the CO_2_ adsorption properties of titanium dioxide through various methods, such as surface functionalization and morphological engineering [6]. Among these, doping TiO_2_ with foreign elements has emerged as a promising strategy. Such a doping can cause a creation of Ti^3+^ sites, improving the photocatalytic performance of titania, as it was proven by Wei et al. in doping with hydrogen and nitrogen [7].

Carbon materials are known as excellent CO_2_ sorbents [8,9,10,11,12,13,14,15,16,17,18] because of their high surface area, microporosity, and mechanical and chemical stability. Among carbon materials, remarkable adsorption properties towards carbon dioxide have been reported for carbon spheres [19,20,21,22].

A high adsorption selectivity to CO_2_ over N_2_ was demonstrated for titania/carbon spheres composites [23], which is of crucial importance, because nitrogen is the main component of flue gases. These nanocomposites also exhibited excellent cyclic stability checked over 10 consecutive adsorption–desorption cycles. 

As is well known, non-metallic doping offers significant advantages over metallic doping in terms of higher photostability, lower cost, and reduced secondary environmental pollution. The use of non-metallic elements such as B, N, C and S, either individually or in combination, to narrow the bandgap and surface state modification of pristine titanium dioxide enhances its photocatalytic performance. For instance, pioneering research by Liu et al. demonstrates that the co-doping of B and N not only increases visible light absorption but also reduces the recombination of photogenerated electrons and holes. This is achieved by forming Ti-O-B-N molecules, which enhance the efficiency of electron transfer, thereby improving photocatalytic degradation under visible light irradiation [24]. They also propose that the introduction of interstitial boron in the TiO_2_ lattice effectively weakens nearby Ti–O bonds, making it easier for nitrogen to substitute for oxygen, thereby increasing visible light absorption and enhancing the chemical stability of doped TiO_2_ [25]. Additionally, Li and colleagues have prepared B and N co-doped black TiO_2_ using a simple sol–gel method combined with magnesium thermal reduction, resulting in stronger light absorption and higher hydrogen production in photocatalysis [26]. However, these studies typically involve a two-step doping process using costly and toxic precursors, inevitably leading to complexity, high costs, and poor production safety.

Among the different dopants to titania, carbon stands out as a particularly attractive candidate due to its unique electronic structure and potential to introduce defect sites within the TiO_2_ lattice. The role of carbon in titania–carbon composites is not only to enhance carbon dioxide adsorption, but also to improve prevention of electron-hole pairs recombination, thanks to the existence of electron scavenger carbon doped into TiO_2_. The enhancement of photocatalytic activity can be attributed to either the band gap narrowing [27] or the formation of localized mid-gap state [28] in TiO_2_ band gap. Table 1 shows recent advances in photocatalytic CO_2_ reduction catalysts.

Janus et al. prepared C-doped titania via ethanol carbonization at different temperature [34] from 150 to 400 °C or at 180 °C under the pressure of 10 bar [35]. With increasing carbon content in TiO_2_ photocatalysts, the activity for phenol decomposition under UV light decreased, but that under visible light was stable. The photocatalytic activity of the commercial titania P25 modified under pressure with ethanol, tested during three azo dyes decomposition under UV light irradiation, was two times higher than for unmodified titania.

Kang et al. [36] described visible sensitive carbon-doped TiO_2_ photocatalyst via grinding titania with ethanol and heating treatment. The formation of Ti–C and C–O bindings in the ground product was determined by heating at 200 °C. Further heating the product at 400 °C caused a decrease in the photocatalytic activity because of the dissociation of Ti–C bindings in the product, whilst the C–O bindings were still maintained.

The composites of titania with carbon spheres used for the reduction in CO_2_ has been studied within the project PhotoRed [37,38]. 

**Figure 1:**
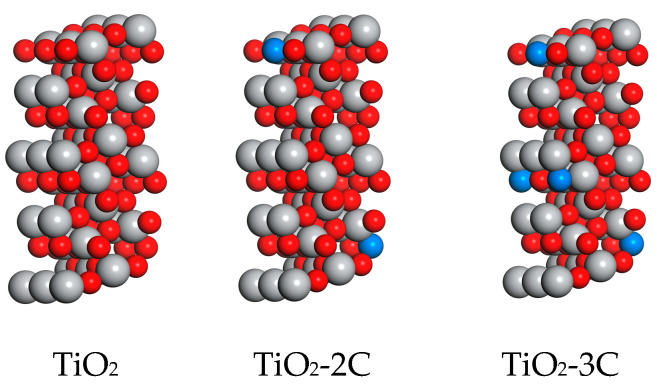
Microstructure of TiO_2_ and TiO_2_ doped with different concentrations of C (the gray sphere represents Ti, the red sphere represents O, and the blue sphere represents C).

This article employs the Density Functional Theory (DFT) to investigate the impact of carbon (C) doping on titanium dioxide (TiO_2_). The created TiO_2_ structures consist of 48 atoms (32 oxygen atoms and 16 titanium atoms). To simulate the influence of different carbon concentrations on the crystal structure, researchers have created two different carbon doping levels (4% and 6%), referred to as TiO_2_-2C and TiO_2_-3C, respectively. The models for all structures are depicted in Figure 1. Subsequently, the electronic density of states (DOS) distribution for these different structures is studied to explore the interactions between individual atoms. The analysis of the DOS sheds light on the electronic structure modifications induced by C doping. Furthermore, the free energies of each structure were evaluated, and the energy requirements at each stage of CO_2_ adsorption were analyzed. This enabled a deeper understanding of the role of TiO_2_ in the CO_2_ adsorption process, elucidating its impact on CO_2_ adsorption capacity.

## 2. Materials and Methods 

All the computational simulations were conducted within the framework of Materials Studio, utilizing the CASTEP and DMol^3^ modules to explore a wide range of surface models of titanium dioxide. These simulations aimed to investigate the structural and electronic properties of TiO_2_ surfaces under various conditions. To avoid unwanted periodic interactions, a 20 Å vacuum layer was carefully introduced around the surface models. In the calculation of the band gap, the Perdew–Burke–Ernzerhof (PBE) approximation within the Generalized Gradient Approximation (GGA) method was employed [39]. However, in the case of transition metal oxides, the GGA approach may introduce self-interaction errors, affecting the electronic structure. To mitigate this error and alleviate the underestimation of the band gap and the delocalization of electrons, the GGA+U method was employed. Specifically, the 3D orbitals of Ti were treated with an additional Coulomb potential (U_Ti_ = 8) to account for the strong on-site electron–electron interactions [40]. This enabled a more accurate representation of the TiO_2_ electronic structure and its response to different surface modifications. In terms of computational parameters, a plane wave energy cutoff of 400 eV was chosen to ensure a well-converged solution for the electronic states. Additionally, norm-conserving pseudopotentials were employed to efficiently describe the interaction between valence electrons and atomic cores. The Brillouin zone integration was performed using a 1 × 2 × 1 Monkhorst-Pack grid, which provided an adequate sampling of the reciprocal space. To obtain reliable structural and energetic properties, extensive structural optimizations were conducted until the forces acting on each atom were minimized to a convergence criterion of less than 10^−3^ eV Å^−1^. This ensured that the simulated systems reached their most stable configurations and allowed for accurate comparisons between different surface models.

After structural optimization, the lattice parameters of pure rutile-type TiO_2_ were calculated to be a = b = 7.5520 Å and c = 18.972 Å, which closely match experimental results [41]. This demonstrates the reliability of the computational methods and results presented in this study.

For pure TiO_2_, the calculated average Ti–O bond length was found to be 2.055 Å. The results are as expected [42]. The doped TiO_2_, following geometric optimization, is summarized in Table 2. It can be observed from the table that the average C-O-Ti bond length in the doped superlattice shows significant variations. This indicates that the introduction of C elements induces pronounced lattice distortion, facilitating easier electron transitions from the valence band to the conduction band.

## 3. Results and Discussion

**Figure 2:**
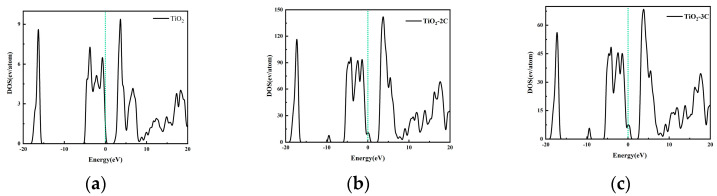
Total DOS of (**a**) TiO_2_ (**b**) TiO_2_-2C and (**c**) TiO_2_-3C. The green dashed line represents the Fermi energy level.

**Figure 3:**
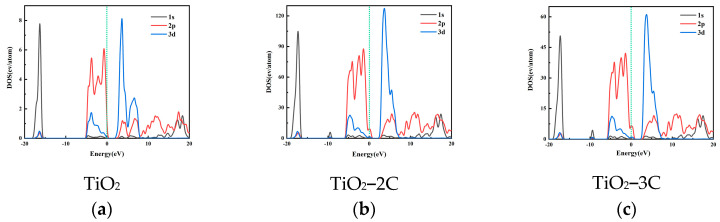
PDOS of (**a**) TiO_2_ (**b**) TiO_2_-2C and (**c**) TiO_2_-3C. The green dashed line represents the Fermi energy level.

The electronic band density has multiple impacts on the CO_2_ reduction reaction. Firstly, the material’s electronic band structure determines its ability to effectively supply the necessary electrons to CO_2_ molecules adsorbed on the surface, thus influencing reduction activity. Secondly, a higher electronic band density aids in enhancing material conductivity, facilitating faster conduction of electrons from external sources to the reduction reaction sites, thereby promoting reaction rates. Additionally, different electronic band structures may lead to distinct product selectivities, and tuning the band structure allows for control over product types and proportions. Figure 2 displays the Density of States (DOS) for pure titanium dioxide and carbon-doped titanium dioxide. It is worth noting that the actual band gap of rutile titanium dioxide is 3.23 eV [43]. However, in this simulation, the band gap calculated using the Generalized Gradient Approximation (GGA) method is 2.13 eV. This is due to the lower calculated position of the valence band, resulting in a smaller band gap. Upon carbon doping, the band gaps of TiO_2_-2C and TiO_2_-3C are reduced to 1.58 eV and 1.47 eV, respectively. These findings underscore the significance of optimizing catalytic materials by adjusting electronic band density to promote CO_2_ reduction reactions. To gain a deeper understanding of band gap variations with carbon doping, Figure 3 illustrates the Projected Density of States (PDOS). In the case of rutile-phase titanium dioxide, the valence band is primarily composed of O-2p states, while the conduction band is dominated by Ti-3d states. Following carbon doping, due to lattice distortion, impurity bands above the valence band maximum (VBM) are mainly composed of O-2p states, while the conduction band minimum (CBM) shifts to lower energy levels, facilitating hybridization between C-2p and Ti-3d states [44]. It can be observed that as elemental doping reduces crystal symmetry, the doped energy levels of TiO_2_ with C merge into the band gap above VBM, reducing the energy required for charge carrier transitions and favoring the progressive excitation of electrons from the valence band to the conduction band, thereby facilitating electron transfer.

Subsequently, the adsorption process of carbon dioxide on the surface of titanium dioxide was systematically investigated, and the formation and transformation of intermediates were thoroughly studied. This research holds significant importance for understanding CO_2_ catalytic reduction and the performance of TiO_2_ as a catalyst. Free energy is an important concept used to characterize the stability of a system and the direction of a reaction, representing the energy available to the system. When the system is under constant temperature and pressure, if the Gibbs free energy of the system decreases, it means that the reaction is reversible and proceeds in a more stable direction, while an increase in Gibbs free energy means that the reaction is irreversible and proceeds in an unstable direction [42,45].

The free energy (∆G) was computed using the following formula:

∆G=∆E+ZPE−T∆S

where ∆E means total energy and ZPE was the zero-point energy; the entropy (∆S) of each adsorbed state were obtained from DFT calculation, whereas the thermodynamic corrections for gas molecules were from standard tables.

**Figure 4:**
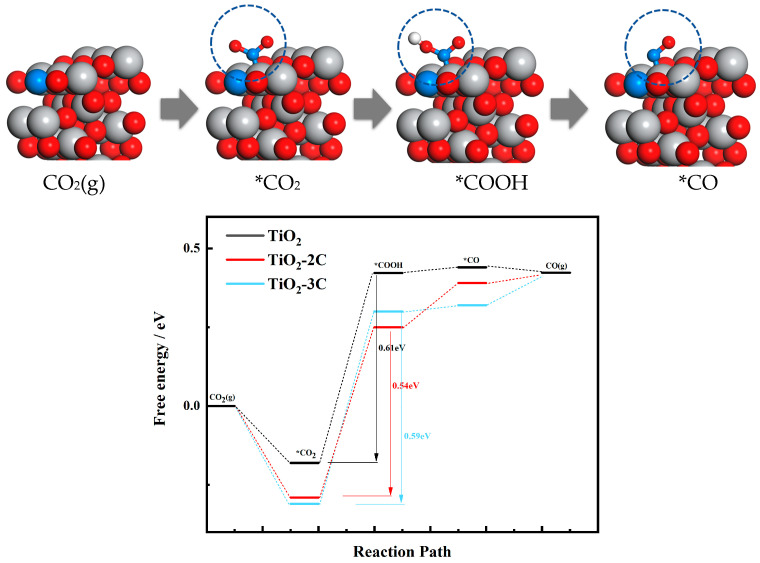
CO_2_ adsorption by TiO_2_ (e.g., TiO_2_-2C) and the free energy required for each structure to undergo the reaction.

Figure 4, exemplifying the TiO_2_-2C configuration, provides a representation of the atomic adsorption process occurring at each stage of CO_2_ reduction. Through the simulation of intermediate structures, it becomes possible to derive the respective free energy changes associated with each of these processes. This comprehensive analysis is of paramount importance in advancing our understanding of CO_2_ reduction mechanisms. Initially, during the carbon dioxide adsorption process, CO_2_ molecules chemically adsorb on the TiO_2_ surface, forming *CO_2_ intermediate species. The activated molecules are denoted by asterisks. This adsorption process releases heat, indicating favorable energy for carbon dioxide adsorption on TiO_2_. This initial adsorption serves as a crucial starting step for subsequent intermediate formation. The results reveal that TiO_2_-2C and TiO_2_-3C require energy of −0.29 eV and −0.36 eV, respectively, for CO_2_ adsorption, both of which are lower than pure TiO_2_ (−0.18 eV). This suggests that TiO_2_ doped with carbon is more prone to CO_2_ adsorption, attributed to its structural bandgap. Enhanced conductivity can provide electrons to CO_2_, allowing it to closely bind to the crystal plane.

Subsequently, hydrogen atoms (H) interact with *CO_2_, forming *COOH intermediates. However, our calculations indicate that the formation of *COOH is endothermic, requiring energy absorption. Thus, *COOH formation might be the energy-limiting step of the entire reaction, influencing the rate and efficiency of the overall process. Additionally, the *COOH intermediate is transient, existing in a higher-energy state during the reaction and likely acting as a temporary reaction intermediate. This process entails overcoming a certain energy barrier and is influenced by reaction temperature, environmental conditions, and catalyst properties. Significantly, the energy barriers for doped TiO_2_ are lower compared to pristine TiO_2_, measuring 0.54 eV and 0.59 eV for TiO_2_-2C and TiO_2_-3C, respectively, compared to the original TiO_2_’s 0.65 eV. This implies that carbon-doped TiO_2_ more readily forms *COOH intermediates, consequently accelerating the reaction process. The introduction of carbon leads to changes in the lattice structure, influencing the adsorption energy and surface diffusion behavior of reactants. These changes likely result in the formation of more stable *COOH intermediates on the TiO_2_ surface, creating favorable conditions for subsequent catalytic steps.

In the subsequent reaction process, *COOH gradually loses its secondary hydroxyl (OH) groups. After dissociation, these OH groups are released from the *COOH intermediate, becoming free hydroxide ions. This state is transient. As *OH further reacts with the catalyst surface, the OH groups on *COOH progressively detach, releasing hydroxyls and forming CO.

In summary, our research provides valuable insights into the complex processes of carbon dioxide adsorption, intermediate formation, and conversion on the surface of TiO_2_. These findings are of significant importance for optimizing the design of titanium dioxide catalysts and enhancing their catalytic ability for carbon dioxide reduction. Understanding these intricate mechanisms will aid in the development and production of efficient catalysts for carbon dioxide reduction. Furthermore, this knowledge can be extended to other catalytic systems, contributing to sustainable energy conversion and environmental preservation. By harnessing the catalytic potential of titanium dioxide and its interactions with carbon dioxide, we can more effectively address global energy and climate challenges.

## 4. Conclusions

This study delves into the influence of C element doping on the CO_2_ adsorption performance of TiO_2_ through DFT calculations. Using TiO_2_-2C as an example, we conducted simulations and found that the inclusion of C significantly enhances CO_2_ reduction capacity of TiO_2_.

By analyzing the DOS distributions of each structure, we have unveiled the intricate and complex interactions between C elements and TiO_2_, resulting in profound changes in the electronic structure. The introduction of C elements triggers a significant distortion in the TiO_2_ lattice. Notably, the band gaps of TiO_2_-2C and TiO_2_-3C have been dramatically reduced from their initial values of 2.13 eV (for pure TiO_2_) to a mere 1.58 eV and 1.47 eV, respectively. This reduction in band gaps holds the potential to substantially enhance the efficiency of TiO_2_ as a catalyst for carbon dioxide conversion. Furthermore, the outcomes of our free energy analysis prominently highlight the paramount impact of carbon doping on titanium dioxide. The formation of the *COOH intermediate, known to be an endothermic process, plays a pivotal role in dictating the reaction rate. In comparison, carbon-doped titanium dioxide experiences a significant reduction in energy barriers, measuring only 0.54 eV and 0.59 eV, respectively, in stark contrast to the pristine material. This substantial decrease in energy barriers greatly promotes the formation of *COOH intermediates during the carbon dioxide conversion process. As a result, the reaction rate is accelerated, potentially leading to a significantly improved efficiency in the carbon dioxide conversion of carbon-doped titanium dioxide.

The findings of this research offer crucial guidance for designing optimized CO_2_ capture materials. C element doping not only improves TiO_2_’s adsorption capability but also opens new possibilities for future carbon capture technologies. These achievements are of vital significance in addressing global carbon emissions and promoting sustainable development. Future research could further explore the CO_2_ adsorption mechanisms of TiO_2_ under various doping conditions, expanding its application range and achieving more efficient CO_2_ capture materials.

## Figures and Tables

**Table 1:** Recent advances in photocatalytic CO_2_ reduction catalysts.

Catalyzer	Main Products	Product Yield	Selectiveness	Ref
Au/TiO_2_	CH_4_	19.7 µmol/m^2^	35	[29]
Cu_1_/TiO_2_	CH_4_	1416.9 ppm g^−1^ h^−1^	/	[30]
Cu/TiO_2_-2/24	CO	1.5 μmol g^−1^ h^−1^	/	[31]
Pt–Au/R-TNTs	CH_4_	28.8 μmol g^−1^ h^−1^	/	[32]
W_18_O_49_@Co	CO	21,180 μmol g^−1^ h^−1^	89.5	[33]

**Table 2:** Average bond lengths (Å) of the doped TiO_2_ after geometry optimization.

Bond Length	Ti–C	C–O	Ti–O
TiO_2_	/	/	2.055
TiO_2_-2C	4.392	3.486	2.622
TiO_2_-3C	4.049	3.162	2.731

## Data Availability

The data that support the findings of this study are available from the corresponding author upon reasonable request.
